# Preventive Effect of a 7-Week App-Based Passive Psychoeducational Stress Management Program on Students

**DOI:** 10.3390/bs14030180

**Published:** 2024-02-25

**Authors:** Elisabeth M. Weiss, Siegmund Staggl, Bernhard Holzner, Gerhard Rumpold, Verena Dresen, Markus Canazei

**Affiliations:** 1 Department of Psychology, University of Innsbruck, 6020 Innsbruck, Austria; siegmund.staggl@uibk.ac.at (S.S.); verena.dresen@uibk.ac.at (V.D.); markus.canazei@uibk.ac.at (M.C.); 2 Department of Psychiatry, Psychotherapy, Psychosomatics and Medical Psychology, University Hospital of Psychiatry II, Medical University of Innsbruck, 6020 Innsbruck, Austria; bernhard.holzner@tirol-kliniken.at (B.H.); gerhard.rumpold@tirol-kliniken.at (G.R.)

**Keywords:** passive psychoeducation, web-based intervention, emotion regulation, coping skills

## Abstract

Passive psychoeducation is an easily accessible and cost-effective self-guided intervention that does not use elements of active psychotherapies or require homework. The present study aimed to investigate the acceptability and efficacy of a 7-week app-based passive psychoeducation stress management program to promote adaptive emotion regulation and coping skills in university students (i.e., 80% psychology students). Participants were tested via Lime-Survey^®^ at pre- and post-test with the Depression Anxiety Stress Scale-21 (DASS-21), the Response Styles Questionnaire (RSQ), and the Emotion Regulation Questionnaire (ERQ). A stratified permutation block randomization by age, gender, and the DASS-21 stress subscale was performed. Each week, the psychoeducation group (*n* = 123) received different psychoeducation modules. At the end of each module, participants answered questions about their satisfaction with each module and adherence to psychoeducation. The control group (*n* = 130) received no intervention. The psychoeducation program led to a significant improvement in the adaptive emotion regulation strategy: “reappraisal” (*p* = 0.004) and a significant reduction in the dysfunctional coping style: “symptom-related rumination” (*p* = 0.01) but not to a significant reduction in depression, anxiety, and stress scores compared to the control group. Thus, the present study might demonstrate a preventive effect of an app-based passive psychoeducation program in students with low clinically relevant psychopathological symptoms.

## 1. Introduction

Stress is an important risk factor for mental and physical health [1,2]. A wealth of empirical evidence suggests that stress increases the likelihood of developing mental illness, particularly depression, and also increases mortality rates, such as suicide [3]. Students in particular are exposed to a variety of stressors. Academic stressors include regular performance reviews, work overload, competitiveness, and worries about the future. Non-academic stressors such as leaving the familiar living environment, finding one’s identity, adapting to a new social environment, and financial stress also play an important role [4,5]. Even before the COVID-19 pandemic, studies showed that students experienced an increased level of stress [6,7,8,9]. Furthermore, the prevalence of mental illnesses among university students, particularly depression and anxiety disorders, increased from 22% to 36% between 2007 and 2017 [10]. In addition to the pre-existing stressors that affect students, the outbreak of the COVID-19 pandemic brought additional stress factors. One such factor was the lack of social contact due to e-learning, which has led to increased anxiety and stress, concentration problems, as well as depressive symptoms and sleep problems [11,12,13,14,15].

Despite the high prevalence of mental illness among university students, approximately two-thirds do not receive adequate psychological help [16,17]. Furthermore, untreated mental health problems among students have a significant negative impact on their quality of life and increase the risk of dropping out of university [18,19,20]. Therefore, the need for easily accessible psychological therapies to help students cope with stress and promote their mental health is significant [21]. In conjunction with establishing therapeutic support services, the implementation of preventative measures that promote students’ stress management and resilience skills should be a top priority for higher education institutions. Preventive stress management is described as an intervention that provides didactic knowledge, including information about stressors, coping strategies, and their application before stressful scenarios occur. In this way, certain behaviors and maladaptive thinking patterns can be improved [22].

According to Romano [23], stress management programs should include three elements. First, objective information about stress and the associated physiological response should be provided. Therefore, it is important to introduce stress modulation techniques, including breathing techniques. Second, because of the various stressors students are exposed to, knowledge of dysfunctional thoughts, emotion regulation, and coping skills plays an important role in psychoeducational stress management programs. These skills have a mediating effect on the health consequences associated with stress [24,25]. The goal is to raise students’ awareness of the role of dysfunctional thinking in the development and maintenance of stress. In addition, students should be introduced to the primary methods of emotion regulation (cognitive reappraisal and expressive emotional suppression) and encouraged to reflect on their own coping behaviors. The third aspect of psychoeducation for stress management focuses on lifestyle habits, especially on raising awareness of healthy and unhealthy habits and educating individuals about the importance of sleep, nutrition, and physical activity.

Today, psychoeducation is an essential component of a comprehensive treatment approach for all mental illnesses. It includes the teaching of disorder-specific information and priorities in both a curative and preventive context. Psychoeducation is based on the principle that knowledge about mental illness and its causes and effects can influence people’s behavior, particularly when they are confronted with stressors [26]. In academic research, a distinction is made between active and passive psychoeducation programs. 

On the one hand, active psychoeducation programs refer to a therapeutic setting with guidance and specific interventions, such as those found in group therapy [27]. Numerous studies suggest that active psychoeducation programs are effective in treating a variety of mental disorders, including anxiety disorders, depression, or chronic pain disorders, but can also reduce caregivers’ burden (for a meta-analysis, see [27,28,29,30,31]). In addition, previous research has shown that active psychoeducational stress management training is also a successful intervention for university students to reduce stress, depression, and other psychological symptoms, although the effect size is rather small (e.g., meta-analyses and reviews by [32,33]).

Passive psychoeducation, on the other hand, is an easily accessible and cost-effective intervention that disseminates information via flyers, the internet, and personal contacts, without using elements of active psychotherapies (e.g., cognitive behavioral therapy [CBT]) or requiring homework such as relaxation exercises that participants perform at home. A meta-analysis of passive psychoeducational interventions showed a small effect on depressive and psychological distress symptoms [27]. 

Internet-based forms of psychoeducation and stress management programs are particularly suitable for younger generations, such as students, who have a firm grasp on digital technologies. App-based stress-management programs offer easy and anonymous access, combined with cost-effectiveness, economy, and flexibility [34,35,36]. Previous meta-analyses have summarized the effectiveness of internet interventions for a variety of mental disorders (e.g., [36,37,38,39]). As a result, increasing attention has been paid to the prevention of mental disorders in recent years. Numerous randomized controlled trials (RCTs) and meta-analyses summarize the effectiveness of a variety of internet-based psychological and psychoeducational interventions in the prevention of various mental disorders (for an overview and meta-analysis, see [40,41,42]). Nevertheless, only a few studies examined the effectiveness of internet-based preventive interventions to promote and develop strength and resilience in younger people with less severe psychopathological symptoms [43,44,45,46,47].

So far, Donker et al., [27] have shown that passive psychoeducation programs have a small effect on depressive and psychological stress symptoms, but evidence for the effectiveness of passive psychoeducation as a preventive measure is lacking. Internet-based passive psychoeducational interventions are relatively easy to implement and can be applied immediately to a large population. They could be a good first step for subclinical populations by overcoming traditional barriers to seeking help for stress, but can also be used as prevention and promotion strategies for mental health at colleges and universities.

Therefore, the aim of this study is to investigate the acceptability and effectiveness of a 7-week app-based passive psychoeducational stress management program to promote adaptive emotion regulation and coping skills in an unselected group of university students. We hypothesized that the 7-week app-based passive psychoeducational stress management program would improve students’ adaptive emotion regulation strategies and reduce maladaptive strategies such as “rumination”, “distraction”, and “suppression” compared to the waiting list control group. Additionally, we expected beneficial effects of the 7-week app-based passive psychoeducational stress management program on symptoms of stress, anxiety, and depression compared to the waiting list control group.

## 2. Materials and Methods

Students at the University of Innsbruck (Austria) were recruited via the university’s official student mailing list (for all courses at the university), social media, and in psychology classes. Bachelor students of psychology received course credit for participation in this study. Data were collected at two timepoints (May 2022 and October 2022). Participation in this study was voluntary and informed consent was obtained from all students prior to participation. This study was in accordance with the 1964 Declaration of Helsinki and was approved by the Ethics Review Board of the University of Innsbruck (No. 26/2022).

The present randomized controlled parallel-group study comprised 294 participants [226 female, 65 male, and 3 gender non-conforming/questioning/other students; mean age: 22.4 years (standard deviation: 3.6)]. In the course of this study, *n* = 41 participants were excluded from the data analyses because they discontinued the study or did not complete all questionnaires. Thus, the data of 253 students (194 female, 57 male, and 2 gender non-conforming/questioning/other students) were finally subjected to data analyses (123 from the psychoeducation group and 130 from the waiting list control group).

### 2.1. Measurements

#### 2.1.1. Mental Health

Mental health was assessed using the Depression Anxiety Stress Scale-21 (DASS-21) [48,49]. The DASS-21 is a self-report screening instrument consisting of three subscales with 7 items that assess symptoms of depression (e.g., “I couldn’t seem to experience any positive feelings at all”), anxiety (e.g., “I was aware of dryness of my mouth”), and stress (e.g., “I found myself getting agitated”) in the past week. Each item is rated on a 4-point Likert scale ranging from 0 (“never”) to 3 (“almost always”), with the total score for each subscale ranging from 0 to 21. The three subscales have good psychometric properties, including internal consistency and validity [50]. The convergent and discriminant validity of the DASS-21 are sufficient [50].

#### 2.1.2. Coping Style

The German short version of the Response Styles Questionnaire (RSQ-D) [51] was developed to measure coping styles according to the response styles theory [52]. It is a 23-item scale containing three subscales: symptom-focused rumination (7 items, e.g., “When I feel sad or depressed, I go away by myself and think about why I feel this way”), self-focused rumination (8 items, e.g., “When I feel sad or depressed, I think I won’t be able to do my job/work because I feel so badly”) and distraction (8 items, e.g., “When I feel sad or depressed, I help someone else with something in order to distract myself”). The items are rated on a 4-point Likert scale ranging from 1 (“almost never”) to 4 (“almost always”). There is evidence of sufficient psychometric properties for clinical and non-clinical patients [51].

#### 2.1.3. Emotion Regulation

To assess how emotions are regulated or controlled, we used the German version of the Emotion Regulation Questionnaire (ERQ) [53]. The ERQ consists of 10 items that assess the habitual use of two emotion regulation strategies, namely cognitive reappraisal (6 items) and expressive suppression (4 items). Cognitive reappraisal refers to the ability to change the way someone thinks about potentially emotion-triggering events. This occurs at an early stage of emotional processing (e.g., “When I am faced with a stressful situation, I will make myself think about it in a way that makes me stay calm”). Habitual suppression of emotional expression is the tendency not to show one’s emotions (e.g., “When I am feeling negative emotion, I make sure not to express them”). The items are rated on a 7-point Likert scale (1 = strongly disagree, 7 = strongly agree). Higher scores on the subscales indicate greater engagement in this strategy. The internal reliability is considerable, where Cronbach’s alpha is 0.73 for suppression and 0.79 for reappraisal [54].

#### 2.1.4. Questionnaire about Satisfaction with Each Module and Adherence to Psychoeducation

After each module, we collected participants’ feedback on satisfaction with the individual modules using the following two questions: 1. How helpful did you find the information material this week? 2. How well informed did you feel about the topic? The items are rated on a 4-point Likert scale, ranging from 4 (“very good”) to 1 (“very bad”).

To explore user engagement and adherence to the app-based passive psychoeducational stress management program, we used the following two questions: 

1. Were you able to apply the knowledge you learned in the psychoeducation in everyday life during the last week? 2. Did you feel relieved by using the psychoeducation?

Again, the items are scored on a 4-point Likert scale, ranging from 4 (“regularly” = more than 5 days a week) to 1 (“never”).

For the analysis, we used the mean value of the questions across the modules.

Finally, we asked open-ended questions about additional features that the participants would like to see included in the next iteration.

### 2.2. Procedure

**Figure 1:**
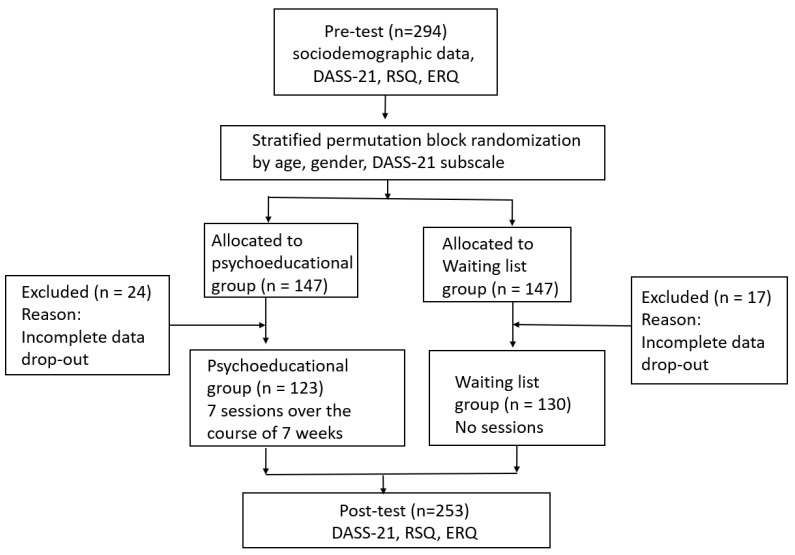
Flow diagram of the present study.

For an overview of the entire study procedure, including the questionnaires used, see Figure 1. The mean values and standard deviations of all study variables are given in the Section 3.

Information on self-reported socio-demographic data was collected at the beginning of this study using a web-based questionnaire. During the 7-week study period, each participant was tested twice, at pre- and post-test, with the following questionnaires: DASS-21, RSQ, ERQ. Psychometric assessment took place online via Lime-Survey^®^. After the pre-test, a stratified permutation block randomization was performed. In a two-stage procedure, all subjects were first grouped into strata (age, gender, and subscale stress score of the DASS-21 at pre-test). In the second step, subjects were assigned to the psychoeducation group (*n* = 147) or the waiting list group (*n* = 147) using block randomization. Over a period of seven weeks, participants in the psychoeducation group received different psychoeducation modules and a push notification on their smartphone every Monday. The order of the modules was fixed and could not be chosen individually by the students (see Table 1). The psychoeducational program was implemented with the software “Quenza App server v. 1.0.0 (Maastricht, The Netherlands)” (https://quenza.com) and all participants used the software via their smartphones. The program comprised seven modules: (week 1) Stress, (week 2) Daily routine, (week 3) Emotion regulation and problem solving, (week 4) Sleep, (week 5) Pleasurable activities, (week 6) Physical activity, and (week 7) Nutrition. Further details on the different modules of the psychoeducational stress management program can be found in Table 1. Each module took approximately 10 to 15 min to complete, but participants in the intervention group were allowed to view the entire content of the modules as many times and for as long as they wished. Researchers monitored each participant’s detailed login information in the backend system and reminded participants via text message to log in and complete the modules. After each module on Friday, we collected satisfaction feedback from the participants and asked open-ended questions about additional features they would like to see in the next iteration. The control group received no intervention but was informed that they would receive access to the full psychoeducation material after the post-test.

### 2.3. Statistical Analyses

In the course of this study, *n* = 41 participants (*n* = 24 in the psychoeducation group; *n* = 17 in the waiting group) were excluded from the data analyses because they discontinued the study or did not complete all questionnaires. Thus, the data of 253 study participants were subjected to data analysis.

Descriptive statistics are presented as means and standard deviations (mean ± SD). To compare demographic variables (age, gender, the number of psychology students per group, psychotherapeutic treatment and mental illness) between the two groups, either a *t*-test, chi-square test, or Fisher’s exact test were performed. Multivariate analyses of variance (MANOVAs) were performed to examine baseline group differences in the questionnaires, using the questionnaire scores as dependent variables. 

A two-way mixed multivariate analysis of variance (MANOVA) was conducted to examine the questionnaire responses, using the between-subjects factor “group” (psychoeducation vs. waiting group) and the within-subjects factor “time” (pre-test vs. post-test treatment week). The group x time interaction was of particular interest as it could reveal the effects of the psychoeducation intervention on the parameters studied. Significant group x time interactions were examined by pairwise post hoc comparisons with Bonferroni correction. We tested for homogeneity of variances in the between-subjects factor using Box’s M test. Furthermore, we examined skewness and kurtosis to test for deviations from normal distribution in the dependent variables. Unless otherwise stated in the Section 3, we confirm that all these assumptions were met. For significant effects, we report partial eta-squared effect sizes. All analyses were conducted with a significance level of 0.05 (two-tailed).

## 3. Results

The age range of the sample was between 18 and 36 years (mean age: M = 22.24 ± 3.23). A total of 194 female, 57 male, and 2 gender-diverse students participated in this study. Of the participants, 86.6% (*n* = 219) were undergraduate students, while 12.2% (*n* = 31) were enrolled in a master’s program and 1.2% (*n* = 3) were students in a PhD program. Most students (80.2%) were studying psychology (*n* = 203).

Regarding mental health, 46 participants (18.2%) reported that they were currently suffering from a mental illness and undergoing psychotherapeutic or psychiatric treatment, and 20.5% (*n* = 52) reported about a history of mental illness and previous psychotherapeutic or psychiatric treatment.

Sociodemographic and study-related information for both groups is shown in Table 2.

At baseline, there were no significant differences between the two groups in terms of the variables age (*t* (251) = −0.94, *p* = 0.35), gender (Fisher’s exact tests with Monte Carlo estimation: *p* = 0.61), the number of psychology students per group (χ^2^ (1) = 1.36, *p* = 0.24), as well as psychotherapeutic treatment (χ^2^ (1) = 2.05, *p* = 0.15) and mental illness (χ^2^ (1) = 0.53, *p* = 0.46).

Based on three separate MANOVAs, there were no significant differences between the psychoeducation group and the waiting list group at baseline in the DASS-21 scales (*F*(3249) = 5.87, *p* = 0.64, ηp^2^ = 0.01), the RSQ-D scales (*F*(3249) = 2.31, *p* = 0.08, ηp^2^ = 0.03), and the ERQ scales (*F*(2250) = 0.01, *p* = 0.99, ηp^2^ < 0.001). The means and standard deviations of the pre-test variables are shown together with the post-test statistics in Table 3.

### 3.1. Mental Health

A multivariate mixed analysis of variance (MANOVA) for the three DASS-21 scales, i.e., depression, anxiety, and stress, showed no significant group x time interaction effect (*F*(3249) = 0.45, *p* = 0.72, ηp^2^ = 0.01) and no main effect of group (*F*(3249) = 0.33, *p* = 0.80, ηp^2^ = 0.004), but a significant main effect of time (*F*(3249) = 5.88, *p* = 0.001, ηp^2^ = 0.07). Post hoc tests showed a significant decrease in both groups only in the DASS anxiety score between the pre- and post-tests (*F*(1251) = 16.56, *p* < 0.001, ηp^2^ = 0.06), but not for the depression (*F*(1251) = 0.94, *p* = 0.33, ηp^2^ = 0.004) and stress (*F*(1251) = 1.11, *p* = 0.29, ηp^2^ = 0.004) subscales.

### 3.2. Coping Style

**Figure 2:**
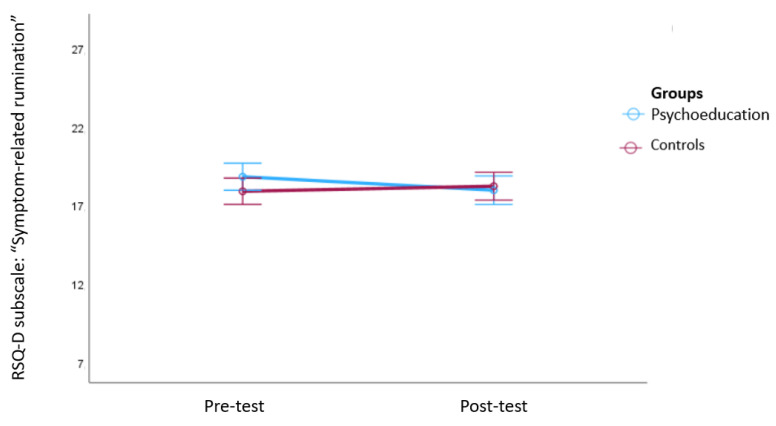
Changes in the RSQ-D subscale “symptom-related rumination” between pre- and post-test. Note: The figure shows means and 95% confidence intervals of the means. A significant decrease in symptom-related rumination only occurred in the psychoeducation group, while the waiting list control group showed an increase.

A multivariate mixed analysis of variance (MANOVA) for the three subscales of the RSQ-D (i.e., symptom-related rumination, self-related rumination, and distraction) revealed a significant interaction of group x time (*F*(3249) = 2.83, *p* = 0.039, ηp^2^ = 0.03) and a significant main effect of time (*F*(3249) = 22.72, *p* < 0.001, ηp^2^ = 0.21). The main effect of group was not significant (*F*(3249) = 0.99, *p* = 0.40, ηp^2^ = 0.01). Post hoc tests showed a significant decrease in the “symptom-related rumination” subscale from the pre- to the post-test phase only for the psychoeducation group, while the waiting list control group showed an increase (*F*(1251) = 6.46, *p* = 0.01, ηp^2^ = 0.03). See Figure 2 for an illustration of this effect. No significant group x time interactions were found for the “self-related rumination” (*F*(1251) = 0.33, *p* = 0.56, ηp^2^ = 0.001) and “distraction” (*F*(1251) = 0.02, *p* = 0.88, ηp^2^ < 0.001) subscales.

However, both groups showed a significant decrease in the coping strategy: “self-related rumination” (*F*(1251) = 4.22, *p* = 0.04, ηp^2^ = 0.02) and an increase in the subscale: “distraction” (*F*(1251) = 62.78, *p* < 0.001, ηp^2^ = 0.20) between the pre- and post-test.

### 3.3. Emotion Regulation

**Figure 3:**
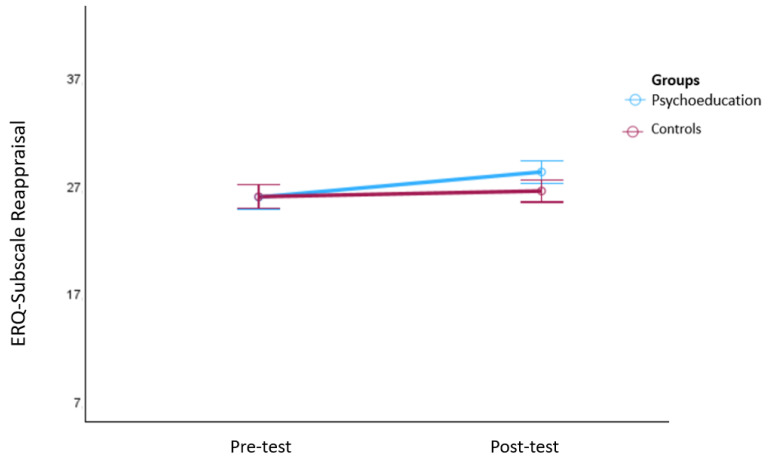
Changes in the ERQ subscale “reappraisal” from the pre- to the post-test phase. Note: The figure shows means and 95% confidence intervals of the means. Significant increases in the subscale: “reappraisal” were only observed in the psychoeducation group.

Finally, a multivariate mixed analysis of variance (MANOVA) was conducted for the “reappraisal” and “suppression” ERQ subscales. The analyses showed a significant group x time interaction (*F*(2250) = 5.08, *p* = 0.01, ηp^2^ = 0.04) and a significant main effect of time (*F*(2250) = 10.45, *p* < 0.001, ηp^2^ = 0.08) but no significant main effect of group (*F*(2250) = 0.89, *p* = 0.41, ηp^2^ = 0.01). Post hoc tests revealed a significant increase in the “reappraisal” subscale from the pre- to the post-test phase only in the psychoeducation group (*F*(1251) = 8.36, *p* = 0.004, ηp^2^ = 0.03). See Figure 3 for an illustration of this effect. No significant effects were found for the “suppression” ERQ subscale (group x time interaction (*F*(1251) = 1.59, *p* = 0.21, ηp^2^ = 0.01) and main effect of time (*F*(1251) = 0.02, *p* = 0.88, ηp^2^ < 0.001).

### 3.4. Satisfaction with and Adherence to the Passive Psychoeducation Program

The three most important topics mentioned by the students were daily structure, stress and regeneration, and sleep. For future adaptations of the program, it was recommended to address topics specific to the target group of students, such as exam anxiety. 

As indicators of acceptability, a questionnaire was used to collect information on satisfaction with the intervention and ratings of the program’s usefulness. Across all modules, students found the modules very helpful (mean ± SD = 2.93 ± 0.46) and they felt well informed about the individual topics (mean ± SD = 3.19 ± 0.45). 

**Figure 4:**
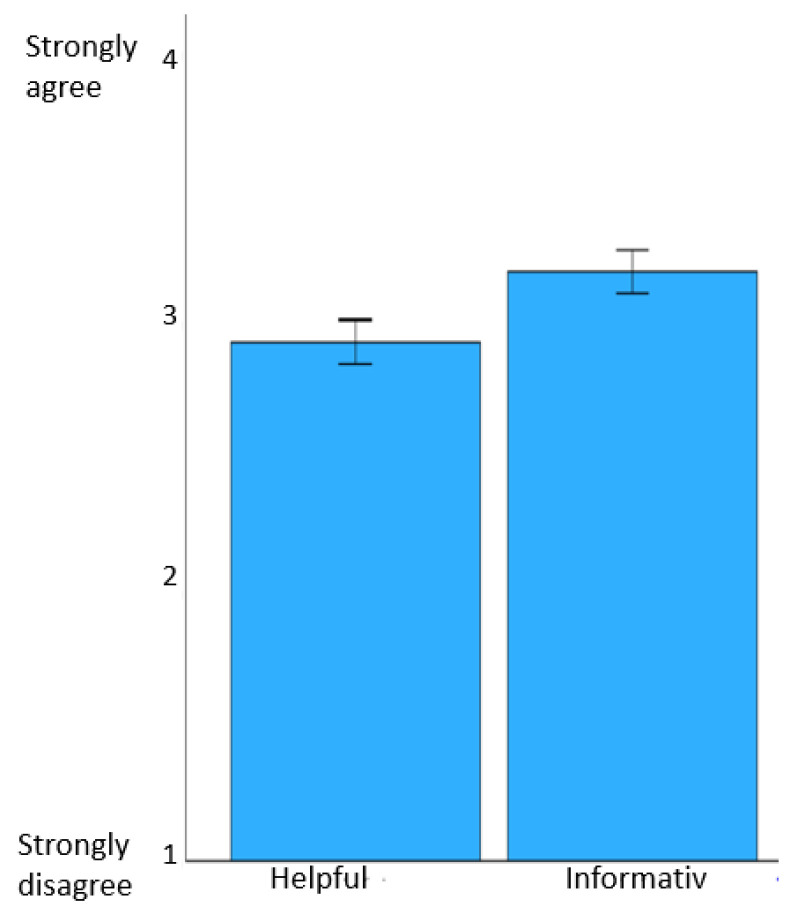
Satisfaction with the modules. Note: the figure shows mean values of questions across the modules and 95% confidence intervals of the means.

**Figure 5:**
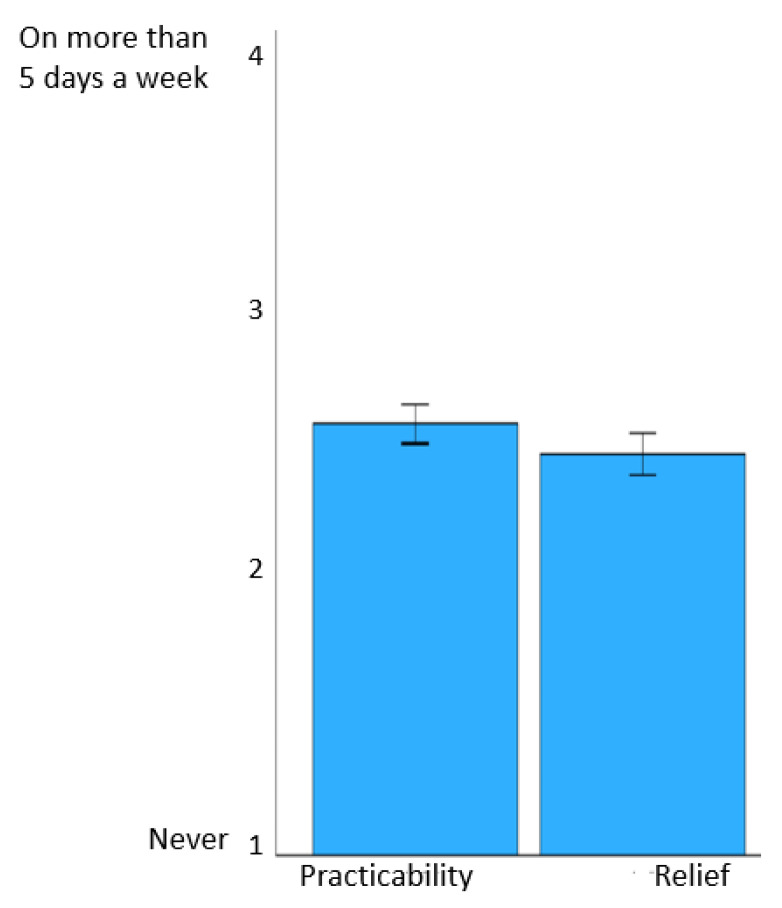
Engagement in and adherence to the psychoeducation program. Note: the figure shows the mean values of questions across the modules and 95% confidence intervals of the means.

On average, they were also able to apply the knowledge they had learned in everyday life on half of the days of the week (mean ± SD = 2.65 ± 0.42) and felt relieved by the use of psychoeducation (mean ± SD = 2.53 ± 0.44). The results with regard to satisfaction with and engagement in/adherence to the program are shown in Figure 4 and Figure 5.

## 4. Discussion

In the present study, a seven-week app-based passive psychoeducation program led to significant improvement in the “reappraisal” adaptive emotion regulation strategy and a significant reduction in the “symptom-related rumination” dysfunctional coping style compared to a waiting list control group. However, the psychoeducation program showed no significant effects on other maladaptive strategies, such as “self-focused rumination”, “distraction”, and “suppression”, compared to the waiting list control group. 

Previous studies with active psychoeducation programs in “face-to-face” settings have shown similar results, demonstrating a significant improvement in adaptive coping styles and a reduction in various maladaptive coping styles [32,55,56,57]. However, it is important to keep in mind that most active psychoeducation programs had a specific focus on training adaptive coping strategies during the courses.

In addition, studies using internet-based active stress management training also showed a positive effect on individual emotion regulation strategies [58,59,60,61,62]. Most internet-based active stress management courses offered additional online support from a trained e-coach to increase adherence to and the effectiveness of the intervention [63,64]. The extent of therapeutic support varied both in form (written support via e-mail, video consultations, additional group sessions, etc.) and in scope (between 1 and 14 h) between the individual studies. Even though internet-based programs for active stress management require significantly fewer resources than face-to-face interventions, they are still associated with increased time and financial expenditure.

In the current study, we were able to show that even a passive psychoeducation program, which is very time- and cost-effective, leads to a significant improvement in adaptive emotion regulation strategies and a significant reduction in dysfunctional coping styles compared to a waiting list control group. 

Despite the improvement in adaptive emotion regulation and coping skills, the seven-week app-based passive psychoeducation program did not result in a significant reduction in depression, stress, and anxiety scores compared to the waiting list group. This is not surprising as the sample scores for all three scales were in a low, non-clinically relevant range at the start of this study. Additionally, these results are probably attributable to the lack of integration of psychological support sessions aimed at resolving emotional difficulties according to targeted treatments. Previous meta-analyses have shown that psychoeducation programs for stress management with active interventions (e.g., mindfulness-based interventions, CBT), either delivered in face-to-face group settings or internet-based, are an effective intervention for students to reduce stress, depressed mood, or other psychological distress (e.g., meta-analyses and reviews in [32,33]). Similarly, the meta-analysis in [27] showed that passive psychoeducation programs also have a small effect on depression and stress symptoms. However, these studies often selected samples with elevated psychopathological scores at the start of the program. In addition, different methodological approaches, such as focusing psychoeducational content on certain symptoms, such as anxiety symptoms, contribute to heterogeneous study results.

## 5. Limitations

Pre–post studies carry the risk of bias, especially in a university setting. It can be assumed that students have different levels of stress at the beginning of their studies, at the beginning of the semester, or during the examination period. Additionally, there is an ongoing debate regarding the long-term effectiveness and sustainability of these programs (see, e.g., [42,65]). Therefore, a follow-up measurement after a period of several months would be necessary to confirm a long-term preventive effect of the 7-week app-based passive psychoeducation program.

Furthermore, a direct comparison with other studies is difficult as the studies differ greatly in terms of student population, duration of intervention, content of psychoeducation, and questionnaires used. The current study consisted mainly of psychology students. This may limit the generalizability of our results, as the stress experience and coping mechanisms of psychology students may not be representative of the general population, as psychology students may have more knowledge about mental health and stress-coping techniques than the average population. Consistent with this reasoning, psychology students in the present study tended to report less stress and fewer mental health problems than students in other programs. In addition, the gender ratio was not balanced across the sample, as the majority of the students (76.7%) were female. The literature about the mental health of students from different academic disciplines is heterogeneous and several factors may account for inconsistent study results, such as the female ratio in the study population as well as demographic factors (e.g., age, socioeconomic status, geographical location, or cultural influence) and a wide variety of course structures (see, e.g., [66,67]).

Internet-based interventions have numerous advantages, such as cost-effectiveness as well as high accessibility, convenience, and flexibility of use at any time and place compared to traditional in-person interventions. At the same time, however, high flexibility in terms of time and location can increase the susceptibility to various confounding variables that may influence the use of the program and completion of the questionnaire. It is important to keep in mind that in the current study, we had no control over whether the study participants actually read the psychoeducation material carefully. However, as an indicator for user engagement and adherence to the psychoeducational modules, we collected participants’ feedback on how often they were able to apply the knowledge they had learned in everyday life and whether they felt relieved by the use of psychoeducation. On average, on half of the days of the week, the students applied their knowledge and felt relieved by the use of the psychoeducation program.

## 6. Implications for Practice and Research

Psychological counseling centers at universities offer low-threshold help for students [68], and a recent systematic review confirmed the effectiveness of face-to-face and online counseling interventions in improving the mental health of university students [69]. A meta-analysis by Osborn and Saunders [70] showed that a good proportion of students use these services, but some barriers still exist, such as low help-seeking behavior and fear of stigmatization, low mental health literacy, or misconceptions about and lack of trust in counselors [69]. Because of the high prevalence of mental health disorders in students, there is an urgent need to expand these services. Web-based or app-based programs, especially, offer the opportunity to provide large-scale psychological support. In practice, passive app- or internet-based psychoeducation programs could be used as a low-threshold component in multi-level counseling concepts in the college environment. First-year students, in particular, are exposed to various stressors, such as coping with new academic demands and external pressure from family, teachers, and society, as well as the need to constantly adapt to new situations. In order to promote stress management skills early on and increase resilience, our passive psychoeducation program could be used in freshman courses. Low-threshold passive psychoeducation programs are particularly relevant to students who are reluctant to use other forms of treatment, as this program offers anonymity, flexibility, easy and constant digital availability, and is cost-effective. 

This study highlights the need for further research, particularly the inclusion of broader and more diverse samples, especially those with elevated psychopathology scores. It would also be beneficial to conduct follow-up measurements to investigate and improve the short- and long-term effectiveness of the psychoeducation program. The addition of more applied content that can be individually selected by students, such as psychoeducation on psychological problems like depression, anxiety, and substance abuse, as well as the implementation of audiovisual or interactive components such as quizzes and case vignettes could increase the effectiveness of the psychoeducation program.

## 7. Conclusions

Effective stress management through the increased use of adaptive coping strategies is considered a preventive factor that can protect against stress-related illness, unhealthy behavior in response to stress, and the long-term physical and psychological effects of stress (see meta-analyses in [71,72]). Thus, in the present study, the preventive effect of an internet-based passive psychoeducation program on students with low clinically relevant psychopathological symptoms was demonstrated. As the program did not include specific interventions such as mindfulness or CBT exercises, it was also very time- and cost-efficient for the students. This was also reflected in the high level of acceptance of the program. Psychological counseling centers at universities offer low-threshold help for students [68], but only some of the help needed is utilized. In practice, passive app- or internet-based psychoeducation programs could be implemented as a low-threshold component in multi-level counseling concepts in university settings.

## Figures and Tables

**Table 1:** Details about the seven modules of the psychoeducational stress management program.

Module	Content
Stress (week 1)	The first psychoeducation module focuses on stress, including stress reactions and the effects of prolonged stress on the body. It also covers the benefits of recovery for the body and provides a brief introduction to self-management strategies.
Daily routine (week 2)	The second psychoeducation module aims to provide guidance on time management and prioritizing tasks. Effective planning strategies are recommended, e.g., creating to-do lists, analyzing daily performance highs and lows, or creating realistic plans with short breaks before and after each activity.
Emotion regulation/problem solving (week 3)	The third psychoeducation module analyses the importance of emotional self-regulation and emotional skills training. It explains the difference between maladaptive strategies for emotion regulation, such as suppression of thoughts/emotions, avoidance, or rumination, and adaptive emotional skills, such as high emotion recognition skills (e.g., acceptance, reappraisal, focusing on positive aspects of the situation). In addition, the six-step problem-solving model is discussed.
Sleep (week 4)	The fourth psychoeducation module focuses on the importance of healthy sleep–wake behavior and rules for sleep hygiene. It provides information on a range of practices and habits that promote healthy sleep. These include keeping regular bedtimes and wake-up times, avoiding naps, getting enough exercise during the day, and creating a comfortable and peaceful sleep environment.
Pleasurable activities (week 5)	The fifth psychoeducational module informs about the health benefits of pleasurable activities. It lists different types of pleasurable activities and gives instructions on how to plan them effectively.
Physical activity (week 6)	The sixth psychoeducation module focuses on the importance of physical activity for physical and mental health. It contains detailed information on suitable sports and exercises to reduce stress, strengthen muscles, and improve mobility, as well as advice on how to incorporate more exercise into your everyday life.
Nutrition (week 7)	The final psychoeducational module provides an overview of dietary guidelines (e.g., the importance of drinking enough water and opting for vegetables, fruit, whole grains, and low-fat dairy products while reducing sugar and salt intake). The module also provides additional information for vegetarians and vegans as well as various links for meal preparation.

**Table 2:** Sociodemographic and study-related information.

	Psychoeducation Group	Waiting List Control Group	Total
	*n* = 123	*n* = 130	*n* = 253
Age [years]: mean (SD)	22.04 (2.8)	22.42 (3.59)	22.24 (3.23)
Sex			
Female: *n* (%)	95 (77.2%)	99 (76.2%)	194 (76.7%)
Male: *n* (%)	28 (22.8%)	29 (22.3%)	57 (22.5%)
Gender-diverse: *n* (%)		2 (1.5%)	2 (0.8%)
Level of education			
Bachelor students: *n* (%)	103 (83.7%)	116 (89.2%)	219 (86.6%)
Master students: *n* (%)	18 (14.7%)	13 (10%)	31 (12.2%)
PhD students *n* (%)	2 (1.6%)	1 (0.8%)	3 (1.2%)
University courses			
Social sciences Psychology students	10 (8,1%) 95 (77.2%)	8 (6.1%) 108 (83.1%)	18 (7.1%) 203 (80.2%)
Natural science	8 (6.5%)	6 (4.6%)	14 (5.5%)
Medical and health science	1 (0.8%)	1 (0.8%)	2 (0.8%)
Humanities	5 (4.1%)	4 (3.1%)	9 (3.6%)
Engineering and Technology	4 (3.3)	3 (2.3%)	7 (2.8%)
	Mental illness		
Psychotherapeutic treatment	26 (21.1%)	20 (15.4%)	46 (18.2%)
Mental illness	28 (22.8%)	24 (18.5%)	52 (20.6%)

Note: SD = standard deviations.

**Table 3:** Means and standard deviations of pre-test variables.

	Psychoeducation Group		Waiting List Control Group	
	Pre *M (SD)*	Post *M (SD)*	Pre *M (SD)*	Post *M (SD)*
DASS-21				
Depression	5.64 (4.25)	5.45 (4.48)	5.55 (4.21)	5.28 (4.00)
Anxiety	5.10 (3.64)	4.24 (3.33)	4.55 (3.68)	4.02 (3.49)
Stress	7.59 (4.18)	7.33 (4.25)	7.31 (3.84)	7.10 (3.67)
RSQ				
Symptom-related rumination	18.85 (5.05)	18 (5.08)	17.93 (4.58)	18.25 (5.10)
Self-related rumination	16.89 (4.37)	16.60 (4.31)	17.33 (4.19)	16.82 (4.33)
Distraction	17.75 (3.38)	18.15 (3.56)	17.25 (4.29)	17.71 (4.01)
ERQ				
Reappraisal	26.03 (6.11)	28.39 (5.66)	26.08 (6.81)	26.61 (6.17)
Suppression	13.59 (4.96)	13.25 (4.99)	13.65 (4.95)	13.92 (5.17)

Note: The table shows means (*M*) and standard deviations (*SD*). DASS-21: Depression Anxiety Stress Scale-21; RSQ = Response Styles Questionnaire; ERQ = Emotion Regulation Questionnaire.

## Data Availability

The datasets generated and/or analyzed during the current study are available from the corresponding author upon reasonable request.
